# An evaluation of the process of informed consent: views from research participants and staff

**DOI:** 10.1186/s13063-021-05493-1

**Published:** 2021-08-18

**Authors:** Lydia O’ Sullivan, Laura Feeney, Rachel K. Crowley, Prasanth Sukumar, Eilish McAuliffe, Peter Doran

**Affiliations:** 1 School of Medicine, University College Dublin, Belfield, Dublin 4, Ireland; 2 Health Research Board-Trials Methodology Research Network, Galway, Ireland; 3 Department of Endocrinology, Saint Vincent’s University Hospital, Dublin 4, Ireland; 4 University College Dublin Centre for Interdisciplinary Research, Education and Innovation in Health Systems, Belfield, Dublin 4, Ireland

**Keywords:** Informed consent, Clinical research, Clinical trials, Methodology, Surveys and Questionnaires

## Abstract

**Background:**

The process of informed consent for enrolment to a clinical research study can be complex for both participants and research staff. Challenges include respecting the potential participant’s autonomy and information needs while simultaneously providing adequate information to enable an informed decision. Qualitative research with small sample sizes has added to our understanding of these challenges. However, there is value in garnering the perspectives of research participants and staff across larger samples to explore the impact of contextual factors (time spent, the timing of the discussion and the setting), on the informed consent process.

**Methods:**

Research staff and research participants from Ireland and the UK were invited to complete an anonymous survey by post or online (research participants) and online (research staff). The surveys aimed to quantify the perceptions of research participants and staff regarding some contextual factors about the process of informed consent. The survey, which contained 14 and 16 multiple choice questions for research participants and staff respectively, was analysed using descriptive statistics. Both surveys included one optional, open-ended question, which were analysed thematically.

**Results:**

Research participants (169) and research staff (115) completed the survey. Research participants were predominantly positive about the informed consent process but highlighted the importance of having sufficient time and the value of providing follow-up once the study concludes, e.g. providing results to participants. Most staff (74.4%) staff reported that they felt very confident or confident facilitating informed consent discussions, but 63% felt information leaflets were too long and/or complicated, 56% were concerned about whether participants had understood complex information and 40% felt that time constraints were a barrier. A dominant theme from the open-ended responses to the staff survey was the importance of adequate time and resources.

**Conclusions:**

Research participants in this study were overwhelmingly positive about their experience of the informed consent process. However, research staff expressed concern about how much participants have understood and studies of patient comprehension of research study information would seem to confirm these fears. This study highlights the importance of allocating adequate time to informed consent discussions, and research staff could consider using Teach Back techniques.

**Trial Registration:**

Not applicable

**Supplementary Information:**

The online version contains supplementary material available at 10.1186/s13063-021-05493-1.

## Background

Informed consent depends on disclosure of pertinent information, capacity to give consent and a voluntary decision [1, 2]. In the clinical research context, the research participant must freely give their informed consent prior to enrolment onto a clinical trial or research study [3]. While the Participant Information Leaflet (PIL) provides a record of the information disclosed and the informed consent form (ICF) records the participants’ written decision to take part, it is nevertheless recognised that informed consent is a process [4, 5], rather than a single event enabled solely by paper or electronic documents. This process involves the researcher approaching the prospective participant, providing some initial information about the research study, and as this information is processed, responding to questions and adjusting the level of information to fit the needs of the individual. The process of informed consent often involves building rapport and trust with the prospective participant and should respect cultural and societal norms, such as involving family members or friends. Finally, once it is evident that the individual has adequately understood the study or trial, they make an informed choice about whether they wish to participate. If they have decided to participate, the participants typically sign a consent form and are given a copy of the signed form and the study/trial information to take away with them. However, while researchers agree that maintaining participant autonomy and supporting their decision-making is important, much debate remains on how the informed consent process can be measured and optimised [6].

A number of studies report that most research participants are satisfied with the information provided to them [7–9]. However, there is also ample evidence of participants misunderstanding critical information for good quality consent, such as the risks and the methodological design of the study [10–12]. It is also recognised that the process of informed consent is complex; for example, the language used during the informed consent process and in the PIL/ICF documents can be too difficult for participants to understand [13–15]. Furthermore, while it is the researcher’s responsibility to provide the relevant information to the participant, the available evidence suggests that participants’ information needs can vary considerably [16, 17], though the reasons are not fully understood. Research participants are often unwell when enrolling in a study and therefore their capacity to absorb information may be diminished, putting them at greater risk of misunderstanding some element of the research study [10, 18].

The process of informed consent can be challenging for researchers also. Studies aimed at understanding the consent process from the investigators’ perspective report that the lack of time and difficulty communicating complex concepts are barriers when facilitating informed consent discussions [19–21]. Several studies also suggest that investigators felt that there was a conflict between their dual role as both a researcher and a clinician [22–24]—investigators recognised the need to generate data to improve treatments but were also concerned about minimizing risks of experimental treatments to individual participants. Analyses of informed consent discussions and interviews with investigators indicate that investigators seldom confirm a patient’s level of understanding at any point during the conversation [25, 26]. Despite this, it seems that investigators remain concerned or in some cases uncertain about how well their patients have understood the study [7, 23]. Several studies have also indicated that many research staff do not receive training on how to facilitate an optimal informed consent discussion [5, 27, 28]—this may affect the experience for both staff and participant.

In aggregate, the above studies indicate that the process of informed consent is not straightforward and many factors influence both the participant’s and the investigator’s experience. However, few or no studies have sought to quantify how clinical research participants and staff experience the process of consent and the importance of contextual factors such as the time spent, the setting of the informed consent discussion and the timing at which the participant is approached. Given this gap in our understanding, the aim of this study is to describe how participants and research staff experience the informed consent process and the contextual factors that contribute to their satisfaction with the process.

## Methods

### Survey design and piloting

**Fig. 1:**
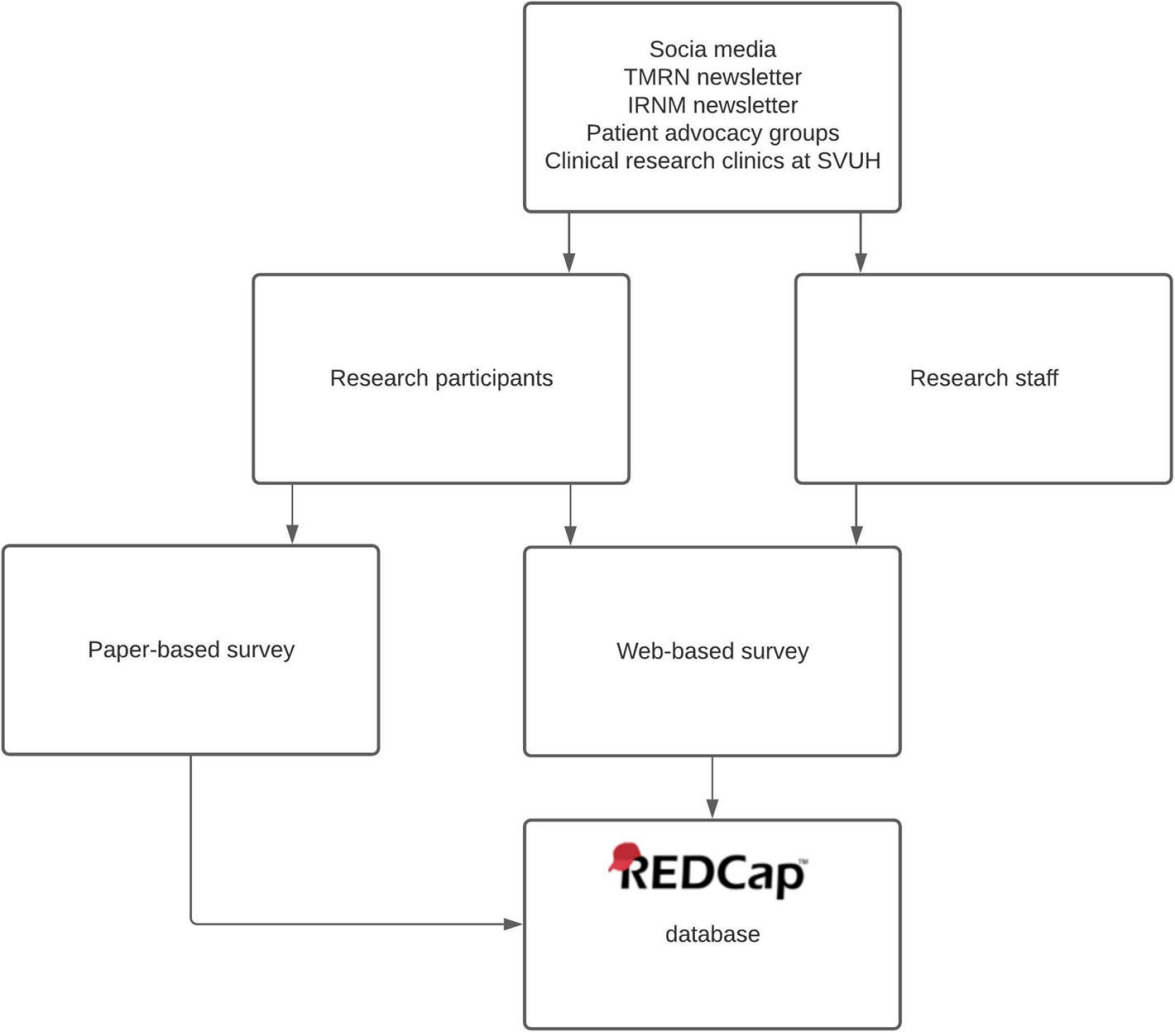
Flowchart describing the dissemination of the surveys. TMRN Trials Methodology Research Network, IRNM Irish Research Nurses and Midwives Network, SVUH Saint Vincent’s University Hospital

This study was conducted in accordance with the principles of the Declaration of Helsinki [29]. Two anonymous surveys were designed to meet the objectives of the study—one for participants (see Additional file 1) and one for research staff (see Additional file 2). The survey for research participants contained 14 multiple choice questions; the survey for research staff contained 16 multiple-choice questions. Both surveys included an optional, open-ended question for respondents to add any additional opinions related to the topic. Figure 1 illustrates how the survey data were collected and stored. For research participants, a matched paper and electronic version of the survey were available. This maximised accessibility for respondents who may not have access to an electronic device or prefer to complete surveys on paper. To allow for participants who had taken part in more than one study or trial, participants were asked to answer the survey based on the most recent study or trial they were consented to. For research staff, an online survey was exclusively used. In order to record their most usual consent practices, research staff were not asked to answer the survey based on their most recent consent discussion, except for one question about the duration of their last consent discussion. Attempts were made to mitigate acquiescence bias (the tendency of responders to provide positive responses) by including Likert scales, neutral questions and options such as ‘I can’t remember’. Both surveys were piloted among six members of the target groups and the wording of the surveys was adjusted following their feedback, to ensure that the questions were clear. Piloting indicated that the surveys took an average of 5 min to complete. Full ethics approval was granted by the Saint Vincent’s Healthcare Group Ethics and Medical Research Committee, Dublin 4, Ireland; Ref: RS20-026. A low-risk ethics exemption was also granted by the University College Dublin Research Ethics Committee; Ref: LS-E-20-117-OSullivan-Doran.

### Sampling

Convenience sampling was used. Research participants were eligible to complete the survey if they were >18 years old and had taken part in a research study in Ireland or the United Kingdom (UK). Research staff were eligible to complete the survey if they facilitate informed consent discussions with an adult, lay research participants in Ireland or the UK. Due to restrictions in place because of the COVID-19 pandemic, some hospital clinics were being conducted by phone, which restricted the distribution of paper surveys. After discussion with the lead research nurse, it was decided that 200 paper surveys could be distributed during the data collection period. Therefore, 200 existing research participants in a single hospital, affiliated with the host university, were offered a paper-based survey by their clinical or research team (research nurse, investigator or clinician) at their routine, in-person, research or clinical visits, with an information leaflet and a stamped addressed envelope to return the survey. This method was used to facilitate participants who prefer a paper-based survey, in order to encourage responses. The surveys were distributed in dermatology, respiratory, oncology, rheumatology, infectious diseases and endocrinology clinics. Requests for research staff were also made via social media (Twitter, Linked In), via the Health Research Board-Trials Methodology Research Network and Clinical Research Coordination Ireland newsletters, and the Irish Research Nurses and Midwives Network. A digital marketing strategy was used to promote the survey to research participants on social media (Facebook, Instagram, Twitter) and through Irish and UK patient advocacy groups. Respondents were also asked to forward the survey to relevant contacts (chain referral sampling). Chain referral sampling provides a swift and cost-effective method of data collection, while ensuring the privacy and confidentiality of prospective respondents [30]. Due to this method of sampling, it is not possible to accurately estimate the response rate for the online version of the survey. However, the response rate was recorded for the paper-based surveys. Responder bias was minimised by using very short [31, 32] and anonymous [33] surveys. Social desirability bias was minimised by ensuring that research participants returned the survey by post and not to their healthcare or research teams, or by completing the survey online. Respondents were advised in the information provided (either in paper version or on the online survey cover page) that by completing and submitting/returning the survey they were indicating their consent to take part (see Additional Files 3 and 4). Data collection took place between September 2020 and February 2021.

### Analysis

Study data were managed using the Research Electronic Data Capture (REDCap) (REDCap 7.4.10, 2019) tool hosted at University College Dublin [34, 35]. For the online surveys, responses were inputted directly into REDCap by the respondents. For the paper surveys, the participants’ responses were manually inputted into REDCap by a single researcher (LOS). The same researcher reviewed the data entry for a random 20% of surveys, selected using Microsoft Excel, after an interval of 3 months to ensure accuracy in data entry.

#### Close-ended questions

Descriptive statistics were used to summarise the following characteristics:

- The research participants’ level of satisfaction with the time, timing, location, level of information and explanation provided
- The research staff’s level of experience, the training provided to them (if any), approach taken when facilitating informed consent discussions, time spent, confidence level, perceived barriers.

Statistical analysis was performed using the Statistical Package for the Social Sciences (SPSS) (IBM SPSS Statistics for Windows, Version 24.0. Armonk, NY: IBM Corp.).

#### Open-ended questions

Responses to the single, optional, open-ended question were extracted—every effort was made to include the *verbatim* text, but where necessary, some details were omitted to ensure confidentiality. The responses were analysed independently using a thematic approach [36] by two researchers (LOS and PS). An experienced qualitative researcher (EMcA) reviewed the extracted codes against the quotations to ensure consistency. The consensus was then reached between the two researchers on the extracted themes.

This study is reported in accordance with the Checklist for Reporting Results of Internet E-Surveys (CHERRIES) [37]—the completed checklist is contained in Additional File 5.

## Results

### Research participants

**Fig. 2:**
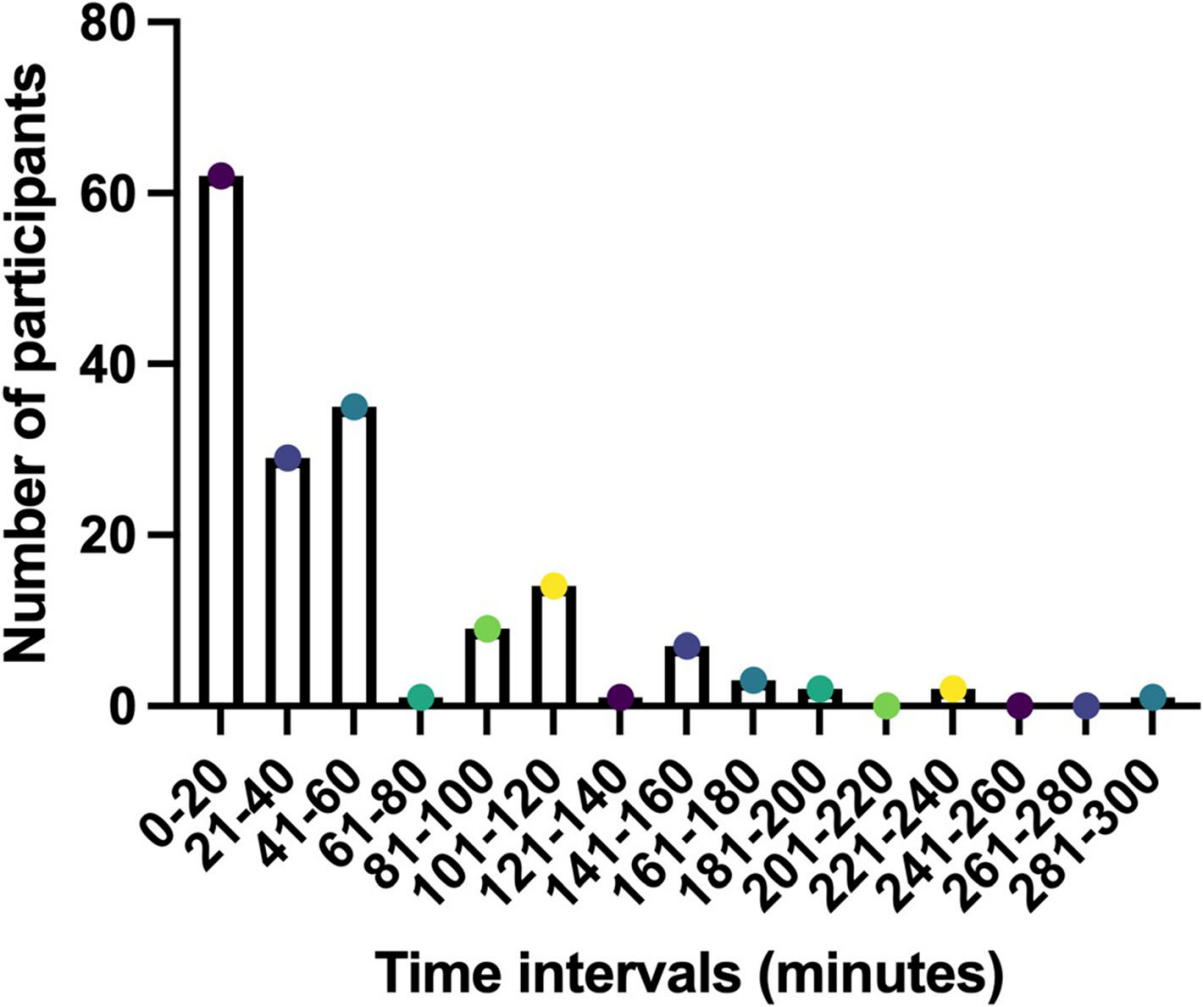
Time taken for informed consent discussion, reported by research participants

One hundred sixty-nine research participants completed the survey. The response rate for the paper-based survey for research participants was 24% (47 out of 200 offered the survey). The remaining responses were to the online survey. Missing fields were denoted in the results by ‘Didn’t Answer’. Table 1 shows the length of time since the participant signed the consent form and whether they felt the location was comfortable and private. Most participants reported having signed the consent form over a year ago (91 or 45%) or in the last year (30 or 18%). Most participants (160 or 95%) felt the location where they signed the consent form was both comfortable and private. In terms of how participants were informed about the study/trial, the majority of participants (137 or 81%) reported that they were given a verbal explanation by the research team, while only 9 (5%) had watched a video or looked at a website about the research study or trial. Overall, 99 (59%) were Very Satisfied and 59 (35%) were Satisfied with their experience of informed consent. The mean time taken for the research participant’s last informed consent discussion was 51 min (range: 1–300 min; median: 30 min)—see Fig. 2.

**Table 1:** Research participant’s experience and satisfaction with the informed consent process

	Number of respondents, ***n***=169 (%)
**How long ago did you sign the consent form?**	
Over a year ago	91 (54%)
In the last year	30 (18%)
In the last few months	15 (9%)
Today	7 (4%)
In the last few weeks	12 (7%)
Cannot remember	14 (8%)
**I felt that the place where the research staff spoke to me was…...**	
Somewhere I felt comfortable and private	160 (95%)
Somewhere I felt uncomfortable or wasn’t private enough	9 (5%)
Before signing the consent form for this study*:	
The research staff explained the study to me	137 (81%)
The research staff gave me an information leaflet to read myself	78 (46%)
The research staff read an information leaflet to me	46 (27%)
I watched a video or looked at a website about the study	9 (5%)
None of these	4 (2%)
Didn’t answer	4 (2%)
**Overall, I was………….with my experience of learning about the research study and signing the consent form**	
Very Satisfied	99 (59%)
Satisfied	59 (35%)
Not satisfied	7 (4%)
Didn’t answer	4 (2%)

*PIL* Participant Information Leaflet; *respondents could select more than one response

Table 2 below indicates that most research participants (149 or 88%) indicated that the time given by the research team to explain the study or trial was about right. The majority of participants also indicated that timing (the day on which they were approached with the study/trial) was a good (74 or 44%) or alright time (41 or 24%). Nearly a third of participants (45 or 27%) responded that the timing would not have made any difference to them. Most respondents (155 or 92%) also felt they had enough time to decide if they wanted to take part.

**Table 2:** Research participants’ views on time and timing of the informed consent process

	Number of respondents (%), ***n***=169
**I feel that the time given by the research staff to explain the study and sign the consent form was:**	
About right	149 (88%)
Not enough	13 (8%)
Too much	5 (3%)
Didn’t answer	2 (1%)
**I felt that the timing (the day and time I was asked to take part in the study) was:**	
A good time	74 (44%)
Timing wouldn’t make any difference	45 (27%)
Alright	41 (24%)
Not the right time: I had heard too much information that day	6 (3.5%)
Not the right time: I was upset or anxious	1 (0.5%)
Didn’t answer	2 (1%)
**I felt that I was given:**	
Enough time to decide if I wanted to take part or not	155 (92%)
Not enough time to decide if I wanted to take part or not	9 (5%)
Didn’t answer	5 (3%)

Table 3 reports how respondents felt about the information, explanation and written information provided to them. The majority of respondents (93%) reported that the amount of information given to them about the study/trial was about right. Similarly, participants said the study/trial was explained to them very well (100 or 60%) or fairly well (58 or 34%). Participants felt that the Participant Information Leaflet was Very Easy (48 or 29%) and Easy (71 or 42%) to understand.

**Table 3:** Research participants’ views on information and explanations given during the informed consent process

	Number of respondents (%), ***n***=169
**I feel that the information I was given about the study was:**	
About right	156 (93%)
Not enough	9 (5%)
Too much	2 (1%)
Didn’t answer	2 (1%)
**I feel that the research staff explained the study:**	
Explained the study very well	100 (60%)
Explained the study fairly well	58 (34%)
Didn’t explain the study well	9 (5%)
Didn’t answer	2 (1%)
**I feel that the research information leaflet and consent form I was given was:**	
Very Easy to understand	48 (29%)
Easy to understand	71 (42%)
Fairly Easy to understand	39 (23%)
Fairly Hard to understand	0 (0%)
Hard to understand	5 (3%)
Very Hard to understand	4 (2%)
Didn’t answer	2 (1%)
**I was:**	
Encouraged to ask questions	140 (83%)
Not encouraged to ask questions	25 (15%)
Didn’t answer	4 (2%)
**My questions:**	
Were answered well	126 (74%)
Were not answered well	6 (4%)
N/A – didn’t have any questions	32 (19%)
Didn’t answer	5 (3%)
**I understood the research study:**	
Very well	76 (45%)
Well	46 (28%)
Fairly well	34 (20%)
Not very well	5 (3%)
Not at all	4 (2%)
Didn’t answer	4 (2%)

One hundred and forty (83%) participants were encouraged to ask questions during the consent process and 126 (74%) felt that their questions were answered well. Most participants were positive about how well they had understood the study/trial: Very Well 76 (45%), Well 46 (28%) and Fairly Well 34 (20%).

#### Optional open-ended question

Seventy research participants responded to the optional, open-ended invitation to add any other feedback. The full list of quotations is included in Additional File 6. The following three themes emerged from these responses:

1. Reports of positive experiences with the research team.
2. The value of allowing sufficient time for the participant to consider the study/trial, including time for questions. This may include providing information to the participant in advance of the clinic visit.
3. The importance of having follow-up after the study/trial has ended, e.g. to be given the results of the trial.

Two sample quotations for each theme are included below:

1.
  - Research participant 022: *‘*[X Nurse], [X Hospital] is excellent, very helpful and so pleasant to deal with. She explains everything clearly and makes sure all your questions are answered. I know she's always on the end of the phone if needed, which gives me great peace of mind. 10/10’
  - Research participant 026: *‘*The research staff were very encouraging and open. I felt very involved in the process’.
2.
  - Research Participant 009: ‘They emailed the document to me before went for study visit. This was really helpful to consider info in my own time in my own surroundings. This meant time could be spent asking for clarification on areas of concern during the meeting without feeling rushed in any way’.
  - Research participant 068: ‘The research study was introduced during a hospital/clinic appointment. I think that a prior notification that this would happen would have been useful. Normally at a hospital appointment, I would already have questions to ask and information to clarify. So the additional information about research can be difficult to process on day. Prior notification would allow the patient time to mentally prepare, and on a practical note allow them to allocate extra time for hospital visit’.
3.
  - Research participant 032: ‘Would like to know how the initial results of the trial going’.
  - Research participant 051: *‘*I would have liked some feedback from the researchers’.

In summary, research participants were positive overall about their experiences of the informed consent process, the time allocated to the process, the amount of information given to them, the environment in which their consent was sought and how well they felt they had understood the study or trial. However, two interesting themes which emerged in response to the open-ended question were the need to allocate sufficient time to the informed consent process and the importance of follow-up or feedback once the trial has finished.

### Research staff

**Fig. 3:**
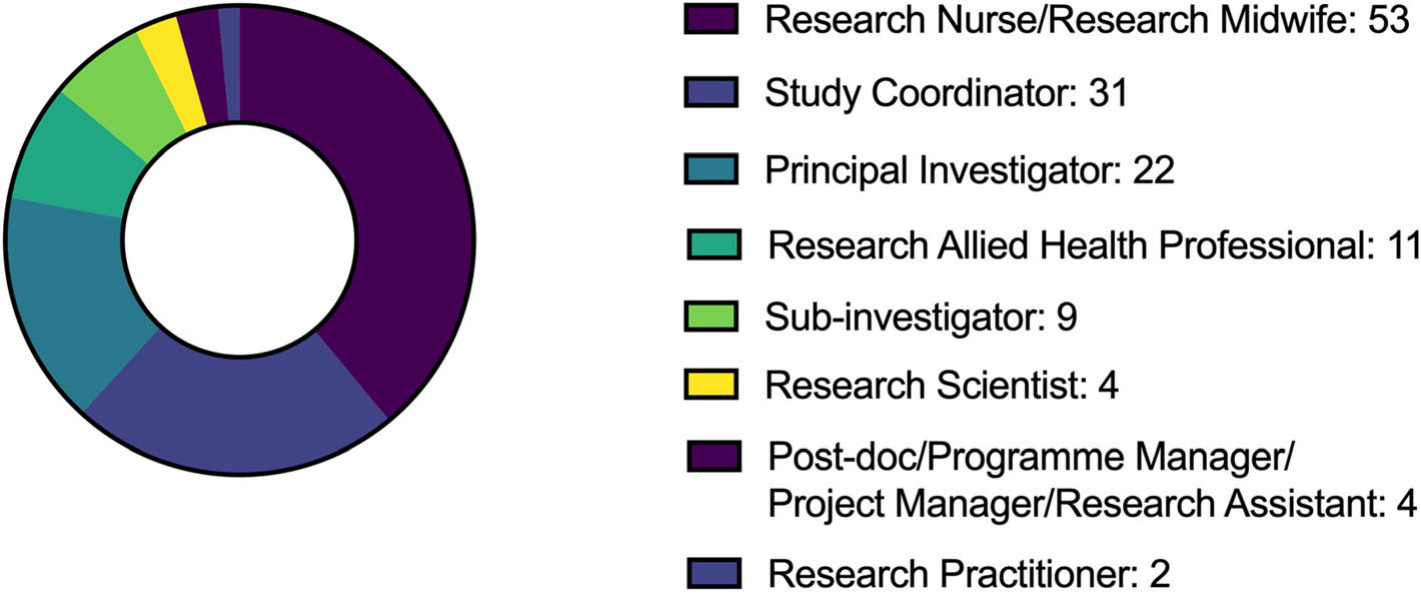
Research staff—roles of respondents (respondents could select more than one response)

One hundred fifteen research staff completed the survey; Fig. 3 and Table 4 describe this cohort. Respondents identified themselves primarily as (respondents could select more than one response):

- Research Nurses or Research Midwives (53 or 46%)
- Study Coordinators (31 or 27%)
- Principal Investigators (22 or 19%).

**Table 4:** Demographics of research staff

**Where do you facilitate informed consent discussions?**	**Number of respondents (%),** ***n*** = 115*
Hospital	99 (86%)
University	15 (13%)
Primary Care/GP	11 (10%)
Telephone	3 (3%)
Community	2 (2%)
**What kind of studies have you/do you work on?**	**Number of respondents (%),** ***n*** = 115*
CTIMPs	83 (72%)
Observational	75 (65%)
Translational/Biomarker/Biobanking	63 (55%)
Registry	40 (35%)
Non-CTIMPs intervention (e.g. surgery, radiation, psychology, physiotherapy, etc. intervention)	45 (39%)
Medical devices	17 (15%)
Nurse-led	1 (0.9%)
Feasibility studies	1 (0.9%)
Non-randomised study of a healthcare intervention	1 (0.9%)
Clinical trial of complex intervention (professional education/medication review)	1 (0.9%)
Qualitative studies	1 (0.9%)
**How much experience do you have facilitating informed consent discussions with clinical research/trial participants?**	**Number of respondents (%),** ***n*** **= 115**
<1 year	10 (9%)
1–2 years	14 (12%)
2–3 years	9 (8%)
3–5 years	13 (11%)
5+ years	69 (60%)
**Have you ever received training on how to take informed consent from a research participant?**	**Number of respondents (%),** ***n*** **= 115**
Yes	74 (64%)
No	38 (33%)
Didn’t reply	3 (3%)
**If yes, was this:**	*n*= 74*
Formal/structured training	45 (58%)
Observation of a senior colleague	41 (55%)
Informal training	39 (53%)
Other	5 (6.8%)

*GP* General practice, *CTIMP* clinical trials of investigational medicinal products, *Post-doc* post-doctoral researcher

*Question had multiple response options

**Fig. 4:**
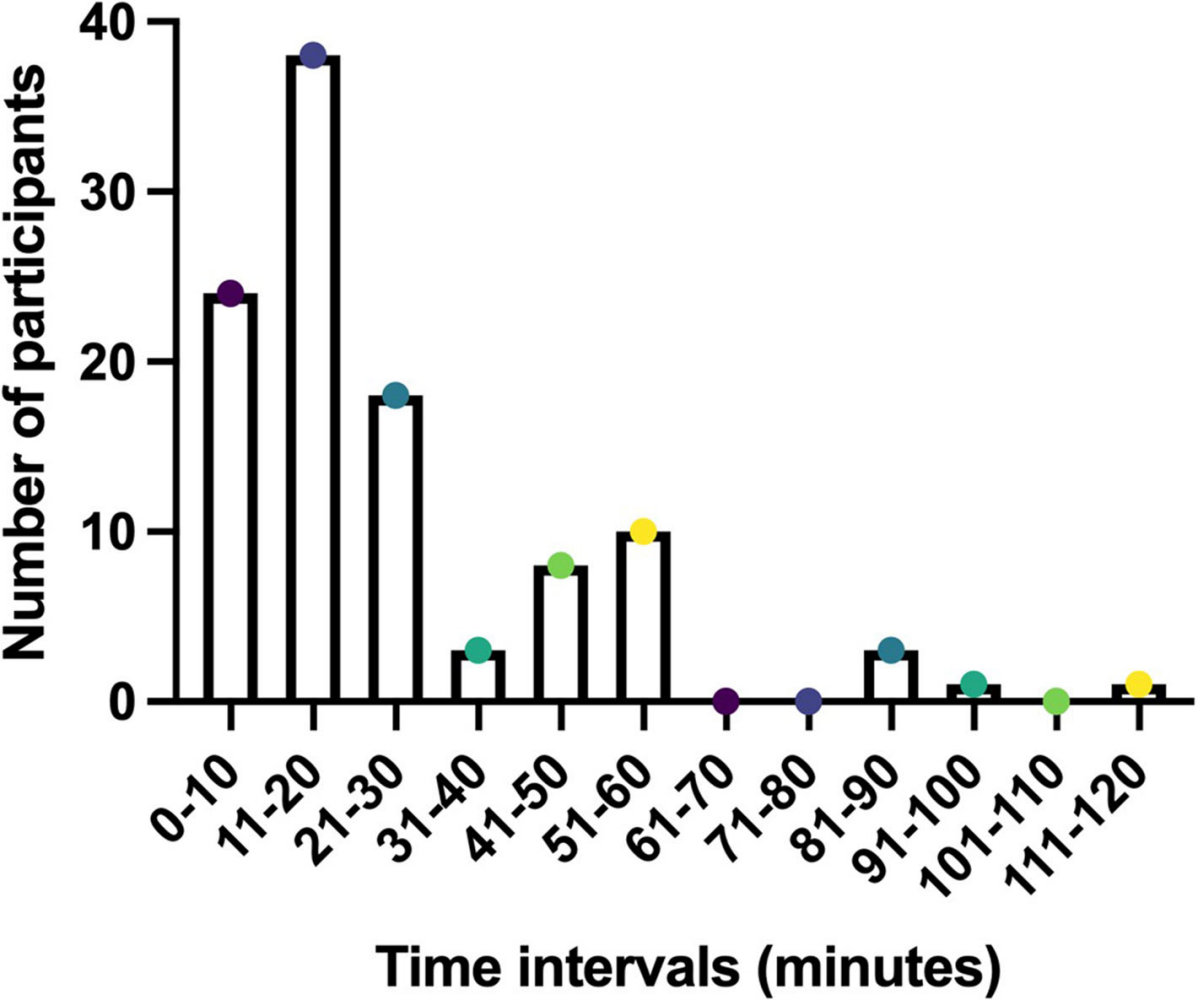
Time taken for informed consent discussion, reported by research staff

The majority of respondents carried out informed consent discussions in hospitals (99 or 86%). Respondents worked on Clinical Trials of Investigational Medicinal Products (CTIMPs) (83 or 72%), Observational studies (75 or 65%), Translational/Biomarker/Biobanking studies (63 or 55%), Registry trials (40 or 35%), non-CTIMP intervention (surgery, psychology, physiotherapy, radiotherapy, etc.) (45 or 39%) and Medical device studies (17 or 15%). The majority of respondents were experienced: 69 (60%) having greater than 5 years and 13 (11%) having 3–5 years of experience facilitating informed consent discussions. Most research staff (74 or 64%) of research staff had received training on how to facilitate informed consent discussions while 38 (33%) had not. Of those who had received training, 45 (58%) had formal/structured training, 41 (55%) observed a senior colleague and 39 (53%) had informal ‘on-the-job’ training (respondents could select more than one answer). Just over one third of research staff (36 or 31%) had received two forms of training while 23 (20%) of respondents had received all three forms of training. The mean time taken during research staff’s last informed consent discussion was 28 min (range: 3–120 min; median: 20 min)—see Fig. 4.

Table 5 reports the difficulties with the informed consent process as reported by research staff. Respondents felt that the PIL/ICF was too long and/or too complex (72 or 63%), the difficulty for participants to understand complex information (64 or 56%), time pressures (46 or 40%), difficulties explaining complex information (44 or 38%), participants being anxious or upset (32 or 28%) and other difficulties (11 or 11%) or no difficulties (4 or 3.5%). In terms of how difficult PILs/ICFs are for participants, 9 (8%) rated them as ‘Very hard’, 44 (39%) as ‘Fairly Hard’, 43 (37%) as ‘Fairly Easy’, 13 (11%) as ‘Easy’, 0 (0%) as ‘Very Easy’ and 6 (5%) did not reply. Research staff reported the following as factors which would deter them from approaching a potential participant:

- Patient is too anxious or upset (55 or 48%)
- Patient does not have enough time (52 or 45%)
- Patient has already received too much information at this visit (44 or 38%)
- Do not think participant will understand the study/trial (32 or 28%)
- Do not have enough time in clinic (31 or 27%)
- Other (21%), did not reply (8 or 7%)
- Not applicable—all eligible patients approached (3 or 2.6%)

**Table 5:** Difficulties reported by research staff with the informed consent process, the level of difficulty of PILs/ICFs and factors which would deter research staff from approaching a participant

**What do you find difficult about facilitating informed consent discussions?**	Number of respondents (%),*n*= 115*
PIL/ICF too long and/or too complicated	72 (63%)
Difficult for patients to understand complex information (disease related or study methodology related)	64 (56%)
Not enough time; time pressures in clinic	46 (40%)
Difficult to explain complex information	44 (38%)
Patient is anxious or upset	32 (28%)
Have dual role of healthcare professional and researcher	26 (23%)
Other:	
Language barrier	2 (1.7%)
Lack of private & quiet space	3 (2.6%)
Participants wanting to please researcher by consenting	2 (1.7%)
GDPR has made PIL/ICF too long and too complex	2 (1.7%)
Other reasons	2 (1.7%)
Not applicable; no difficulties	4 (3.5%)
**Do you think participant information leaflets and consent forms are in general:**	**Number of respondents (%), *n*= 115**
Easy for participants to understand	13 (11%)
Fairly easy for participants to understand	43 (37%)
Fairly hard for participants to understand	44 (39%)
Very hard for participants to understand	9 (8%)
Didn’t reply	6 (5%)
**What situations make you less likely to offer a study/trial to a participant?**	Number of respondents (%),*n*= 115*
Patient is too anxious or upset	55 (48%)
Patient doesn’t have enough time	52 (45%)
Patient has already received too much information at this visit	44 (38%)
Don’t think patient will understand the study/trial	32 (28%)
Don’t have enough time in clinic	31 (27%)
Other	24 (21%)
Didn’t reply	8 (7%)
Not applicable – all eligible patients approached	3 (2.6%)

*Question had multiple response options

*PIL* Participant Information Leaflet, *ICF* Informed Consent Form, *GDPR* General Data Protection Regulation

Table 6 details the approach taken by research staff during the informed consent process: 105 (91%) explain the study verbally, 97 (84%) give participants a PIL and ask them to read it, 55 (48%) read the PIL to participants, 7 (6%) show a participant a video or website and 12 (10%) do ‘Other’, including providing initial information and then following up, working with a translator and summarising important information. Research staff followed a structured approach (i.e., following a checklist or the structure of the PIL): all the time (40 or 35%), often (36 or 31%), occasionally (16 or 14%), never (17 or 15%) and did not reply (6 or 5%). Most research staff (108 or 88%) reported that they check participants’ level of understanding prior to consent, 5 (6%) reported that they did not and 5 (6%) did not answer the question. For those who do check participants’ level of understanding, 88% ask participant if they have understood, 89 (35%) ask the participant to ‘teach back’ or ‘talk back’, 93 (81%) encourage participants to ask questions, 6 (6%) reported using other means, while 2 (2%) did not reply.

**Table 6:** Approach taken by research staff in the informed consent process and confirming participants’ understanding

**Approach taken during informed consent process**	Number of respondents,*n*= 115 (%)*		
Explain verbally	105 (91%)		
Give PIL & ask participant to read it	97 (84%)		
Read PIL to participant	55 (48%)		
Show participant video or website	7 (6%)		
Other	12 (10%)		
**Follow a structured approach (follow a checklist or the layout of the PIL)**	**Number of respondents,*n*= 115 (%)**		
All the time	40 (35%)		
Often	36 (31%)		
Occasionally	16 (14%)		
Never	17 (15%)		
Didn’t reply	6 (5%)		
**Check participant’s understanding**	**Yes**	**No**	**Didn’t reply**
	101 (88%)	7 (6%)	7 (6%)
**If understanding is checked**	Number of respondents (%),*n*= 101*		
Ask participant if they have understood	89 (88%)		
Ask participant to ‘teach back’ or ‘talk back’	35 (35%)		
Encourage participant to ask questions	93 (81%)		
Other	6 (6%)		
Didn’t reply	2 (2%)		
**Continue to monitor consent**			
All the time	30 (26%)		
Often	30 (26%)		
Occasionally	33 (29%)		
Never	16 (14%)		
Didn’t reply	6 (5%)		

*Question had multiple response options

*PIL* Participant Information Leaflet

**Fig. 5:**
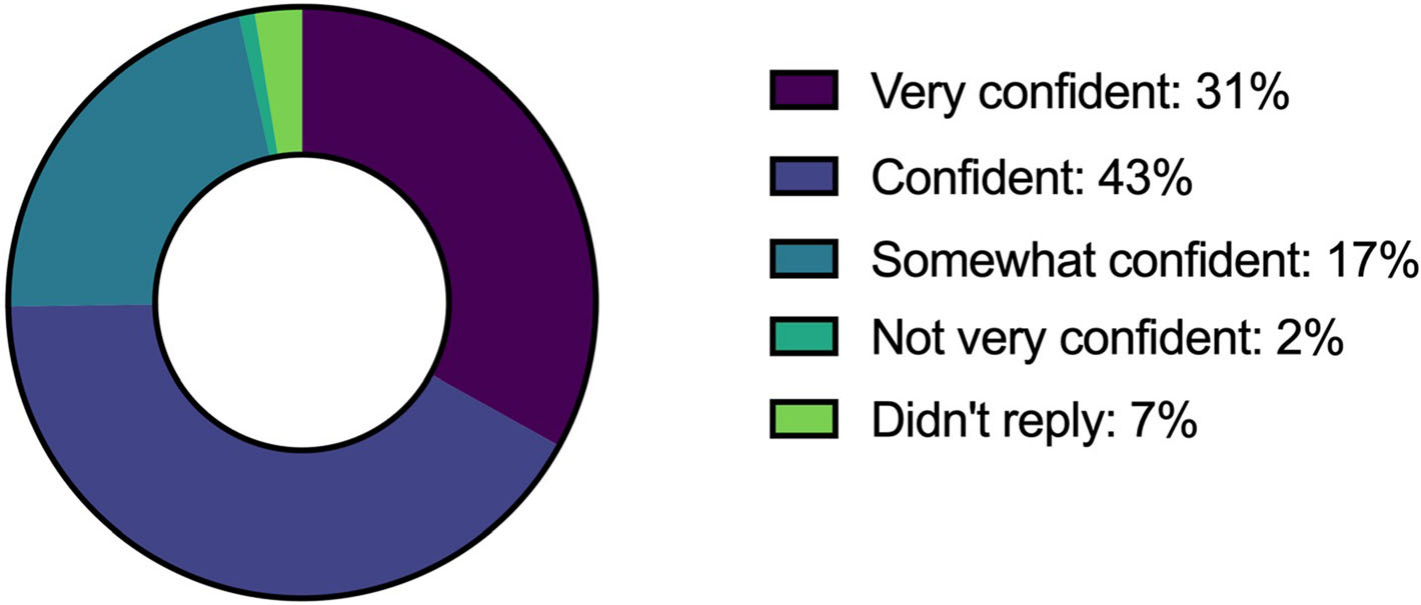
Research staff—confidence level in facilitating informed consent discussions

Figure 5 provides an overview of the confidence level of research staff to facilitate a good informed consent process. Respondents were ‘Very confident’: 36 (31%), ‘Confident’: 49 (43%), ‘Somewhat confident’: 20 (17%), ‘Not very confident’: 2 (2%), ‘Not at all confident’: 0 (0%) and Did not reply 8 (7%).

Table 7 shows the factors which research staff felt would improve the informed consent process for participants: shorter/simpler PIL: (86 or 75%), a PIL with simple diagrams or pictures (68 or 59%), resources like an app or video (56 or 49%), more time with the participant (54 or 47%) and more time with another member of the research team (29 or 25%). Other responses included a quiet, dedicated, uninterrupted space (6 or 5%), PIL with less GDPR information (2 or 2%), PILs in other languages (1 or 0.8%) and miscellaneous (3 or 3%).

#### Optional open-ended question

Thirty research staff responded to the optional, open-ended request to add any other feedback. The full list of quotations is included in Additional File 7. A very dominant theme emerged: research staff feel that the time and resources, particularly space, are important to facilitate a good informed consent process and these factors are often limited in supply. The following sample quotations illustrate this theme:

- Research staff 003: ‘As a researcher, it feels like the definition of informed consent is constantly changing, the bar is always going up. This is of course, a good thing. But the consequence is the need to have ongoing dialogue. This requires significant resources that the system is not currently providing.’
- Research staff 008: ‘Research staff often struggle for dedicated space to conduct informed consent and this can add unnecessary stress and burden to the process. Clinical trials personnel should have dedicated areas for completing this important process with appropriate resources and time availability.’
- Research staff 012: ‘Resources badly needed. Dedicated trial clinics. Protected time.’
- Research staff 015: ‘Discussing consent in a busy clinical environment is very difficult.’

Three additional themes emerged:

1. Consent process can be challenging; training on how to facilitate is needed
2. Technology could be used to improve the informed consent process
3. An accessible PIL/ICF is important; GDPR/data protection information is often too long, too complicated

Two sample quotations for each theme are included below:

1.
  - Research staff 029: ‘I have had many challenging discussions with collaborators around rates of recruitment which may be lower than others but at least I know I am running my studies with the highest ethical standards….it can be very hard though!’

b. Research staff 008: ‘Research staff should have more formalised training in the trial and consent process.’

2.
  - Research staff 005: ‘We need greater facilitation of remote consent (telephone etc.) especially with lack of visiting due to COVID.’
  - Research staff 028: ‘The issue of informed consent, and electronic consent, is increasingly relevant with the COVID-19 pandemic.’
3.
  - Research staff 007: ‘PILs much too complex particularly with data protection which patients find cumbersome and excessive.’
  - Research staff 018: ‘Info leaflets are getting more complicated with GDPR/data protection information. It is almost impossible to make it shorter without risking rejection by ethics committee.’

In summary, research staff in this study reported good levels of experience and confidence in facilitating informed consent discussions. Research staff indicated that a shorter, simpler PIL/ICF or a PIL/ICF with diagrams, the use of technologies and additional time with participants would improve the overall informed consent process.

## Discussion

### Summary of key findings

This study quantified a number of factors which are vital to the informed consent process, from the perspectives of both research participants and research staff. Research participants were generally positive about each aspect of the informed consent process explored in the survey. However, they did highlight the importance of having sufficient time and the importance of providing follow-up once the study/trial concludes, e.g. providing the results to research participants. Research staff reported that they felt quite confident with the process of informed consent overall, possibly reflecting that the respondents in this case were generally experienced in facilitating informed consent discussions. Barriers to the informed consent process noted by research staff included lengthy, complex PILs/ICFs, difficulties communicating complex information and time constraints.

### Participants report positive experience but have they understood?

Research participants in this study overwhelmingly reported a positive experience of the informed consent process and high levels of understanding of the trial or study. This correlates well with previous studies reporting high levels of satisfaction among research participants [9, 38–41]. It is encouraging to report that the majority of research participants are happy with their experience of the informed consent process, which is testament to the dedication and commitment of research staff. However, other studies consistently demonstrate that when research participants are assessed, they often have a poor understanding of key parts of the study or trial [42, 43]. Hietnen’s survey of 261 participants in an oncology trial indicated that while 91% of participants were satisfied with the difficulty level of the information given, 51% had misunderstood randomisation [44]. It is also concerning to note that Pope et al.’s study found that participants who reported to have received the ‘right amount of information’ were unfortunately not found to have a higher level of understanding of blinding [9]. Studies have shown the participants’ information needs vary considerably [16, 17], and it is possible that research participants feel that they have gained sufficient knowledge to make a decision without understanding key aspects of the trial. However, this jars with the doctrine of informed consent, which states that participants must be informed about the pertinent information prior to providing consent [45].

### The importance of adequate time and resources

Research staff consistently reported in this study that they felt that time and resources such as space were limiting factors in their ability to facilitate an optimal informed consent process—40% reported that time pressures were a difficult component of the informed consent process and 47% felt that more time would improve the informed consent process for participants. This finding also emerged in the responses to the open-ended question from both research staff and research participants. Spaar et al. similarly reported lack of time as the biggest barrier to the process of recruitment to randomised trials [20]. This is of concern, since two systematic reviews identified additional time as one of the few factors which has been shown to significantly improve participants’ understanding [46, 47]. Studies by Aaronson [48] and Tindall [49] both found that an additional conversation with a member of the research team improved participants’ understanding. Research participants in this survey study also noted in the open-ended question that it may be helpful to receive the information about a trial in advance of the consent discussion with the research staff, in order to have additional time to consider the information.

### The Participant Information Leaflet/Informed Consent Form

Sixty-three percent of research staff in this study felt that the PIL/ICF is too long and complex and 75% reported that a shorter and simpler document would improve the informed consent process. However, it is interesting that research participants did not share the same view, with the majority stating they felt the PIL/ICF was Very Easy, Easy or Fairly Easy to understand. Lynoe’s and Montgomery’s studies with participants of a chronic dialysis and anaesthesia trial similarly found that most participants found the PIL to be helpful and easy to read [50, 51]. There is evidence that PILs/ICFs are becoming longer and more complex [52, 53], and it is challenging for research teams and sponsors to balance giving the pertinent information without overwhelming potential participants. The General Data Protection legislation introduced in the European Union brought about extra challenges as additional information must now be provided to participants [54, 55]. While participant satisfaction with the information provided is undoubtedly important, helping participants understand the key elements needed to provide informed consent is also critical. Regarding multimedia resources, such as an application, website or video: only 5% of research participants were offered them and only 6% of research staff use them—while further empirical research is needed to assess the effect of multimedia on the quality of informed consent, these kinds of supports may be useful, particularly for some groups of participants [46, 56, 57].

### Teach Back/Talk Back method

In this study, 88% of research staff reported that they confirm a participant’s level of understanding. This self-reported rate was higher than in Jenkins’ analysis of 82 recordings of actual informed consent discussions which indicated that in nearly 83% of cases participant understanding was not confirmed [26]. The majority of research staff in this study reported that they confirm participant’s understanding by asking if they have any questions. However, Nusbaum and colleagues were critical of simply asking the participant if they have understood or if they have any questions—how can a participant judge themselves if they have understood? It may also be difficult for participants to ask questions if they have not understood key pieces of information [28]. In this study, 19% of research participants reported that they did not have any questions to ask. Interestingly, Keller’s semi-structured interviews with 18 research staff indicated that Principal Investigators tend to approach participants who do not ask too many questions and those without a strong personality [42]. Cox noted that 40% of clinical research participants interviewed regarding their experiences did not feel able to ask questions [58]. Thirty-five percent of research staff in this study reported that they used Teach Back/Talk Back strategies to ensure that participants have understood. The Teach Back/Talk Back method involves the patient or service user verbally relaying the information that they have been given back to the provider and has been found to have a positive effect on health communication in the clinical (non-research) setting [59]. Flory and Emanuel’s systematic review of interventions to improve informed consent reported five trials which indicates that test/feedback showed significant improvement in understanding [47]. Perhaps, therefore, Teach Back/Talk Back strategies should be encouraged when researchers are undergoing training on how to facilitate informed consent. However, it should be noted that the Teach Back/Talk Back method may require additional time, which may already be in short supply.

### Limitations

The method of sampling, in particular the use of chain referral sampling is a limitation of this study, as it is non-random in nature [30]. There may have been enthusiasm bias as both research staff and research participants who are interested in optimizing the informed consent process may have been more likely to respond to the survey. Similarly, there may have been selection bias associated with the distribution of the paper surveys to research participants—due to the COVID-19 restrictions, the surveys had to be given out by the research or clinical staff and not systematically by an independent researcher. The survey was developed specifically to fulfil the aims of this research study and was piloted with representatives from both target groups but was not formally validated. It was not possible to estimate the response rate of the online surveys and approximately a quarter of the research participants were recruited from a single hospital—this makes it difficult to assess the generalisability of the results. Structured, anonymised surveys were used to gather quantitative data regarding specific factors which relate to the process of informed consent. While additional open-ended questions may have explored these factors in more depth, previous qualitative studies have investigated the perceptions of research staff and participants [22, 42, 60–62]. It is also important to note that the research staff in this study had a range of experiences, perhaps in different settings, to report on from when answering the survey, while some of the research participants had only a single consent discussion experience to draw from. Finally, the lack of demographic detail for the research participants, e.g. education level, limits the generalisability of the findings of this study.

## Conclusions

Research participants in this study were overwhelmingly positive about their experience of the informed consent process. However, research staff expressed concern about how much participants have understood and studies of patient comprehension of research study information would seem to confirm these fears. Adequate time should be allocated to informed consent discussions and research staff could consider using Teach Back/Talk Back techniques to confirm and enhance understanding.

## Supplementary Information

**Additional file 1.** Research Participant’s Survey.

**Additional file 2.** Research Staff Survey.

**Additional file 3.** Participant Information Leaflet (Research Participants).

**Additional file 4.** Participant Information Leaflet (Research Staff).

**Additional file 5.** Checklist for Reporting Results of Internet E-Surveys (CHERRIES)

**Additional file 6.** Complete list of responses from research participants to open-ended question

**Additional file 7.** Complete list of responses from research staff to open-ended question

## Data Availability

The datasets used and analysed during the current study are available from the corresponding author on reasonable request.

## Abbreviations

GDPR: General Data Protection Regulation
GP: General Practitioner
HRB-TMRN: Health Research Board – Trials Methodology Research Network
ICF: Informed Consent Form
IRNN: Irish Research Nurses and Midwives Network
PIL: Patient Information Leaflet
REDCap: Research Electronic Data Capture
SPSS: Statistical Package for the Social Science
UK: United Kingdom
