# Supplementary material for: An evaluation of the process of informed consent: views from research participants and staff
Source: Trials. 2021 Aug 18;22:544. doi: 10.1186/s13063-021-05493-1 (PMC8371296; doi:10.1186/s13063-021-05493-1)
Supplement: Supplementary file 1 — Additional file 1. Research Participant’s Survey. [file 13063_2021_5493_MOESM1_ESM.pdf]

Research Participant's Questionnaire

Have you taken part in a research study in Ireland or the United Kingdom?

- ☐ Yes
- ☐ No
- ☐

If Yes:

You signed a consent form to take part in a research study in the past. We are interested in what you think about how you were asked to take part in the study and how it was explained to you. We would be very grateful if you could answer the questions below. If you have taken part in more than one research study or trial, please fill out the questions with your **most recent study** in mind. The survey has 14 multiple choice (tick-box) questions. It will take you about 10 minutes to fill in.

1. How long ago did you sign the consent form to take part in a research study?
   - ☐ Today
   - ☐ In the last few weeks
   - ☐ In the last few months
   - ☐ In the last year
   - ☐ Over a year ago
   - ☐ Cannot remember

2. I felt that the place where the research staff spoke to me was:
   - ☐ Somewhere I felt comfortable and private
   - ☐ Somewhere I felt uncomfortable or wasn't private enough

3. About how long did talking about the study and signing the consent form take? Please put in all of the time you spent in the clinic talking about the study with the research staff and signing the consent form. Please include all of the time, even if it was on more than one day – for example, if it was 30 minutes on one day and an hour on another day, please write 1 hour and 30 minutes.

   ________hours ________minutes

4. I feel that the time given by the research staff to explain the study and sign the consent form was:
   - ☐ Not enough
   - ☐ About right
   - ☐ Too much

5. I feel that the timing (the day and time I was asked to take part in the study) was:
   - ☐ Not the right time, I was upset or anxious
   - ☐ Not the right time, I had heard too much information already on that day
   - ☐ Alright
   - ☐ A good time
   - ☐ The timing wouldn't make any difference to me

6. I feel that the research staff:
   - ☐ Didn't explain the study well
   - ☐ Explained the study fairly well
   - ☐ Explained the study very well

7. I feel that the information I was given about the study was:
   - ☐ Not enough
   - ☐ About right
   - ☐ Too much

8. I felt that the research information leaflet and consent form I was given was:
   - ☐ Very easy to understand
   - ☐ Easy to understand
   - ☐ Fairly easy to understand
   - ☐ Hard to understand
   - ☐ Very hard to understand

9. I was:
   - ☐ Encouraged to ask questions
   - ☐ Not encouraged to ask questions

10. My questions:
    - ☐ Were answered well by the research staff
    - ☐ Were not answered well by the research staff
    - ☐ Not applicable – I didn’t have any questions

11. I felt that I was given:
    - ☐ Enough time to decide if I wanted to take part or not
    - ☐ Not enough time to decide if I wanted to take part or not

12. I understood the research study:
    - ☐ Very well
    - ☐ Well
    - ☐ Fairly well
    - ☐ Not very well
    - ☐ Not at all

13. Before I signed the consent form for this study: (Please tick all that apply)
    - ☐ The research staff explained the study to me
    - ☐ The research staff read an information leaflet to me
    - ☐ The research staff gave me an information leaflet to read myself
    - ☐ I watched a video or looked at a website about the research study
    - ☐ None of these

14. Overall, I was _________
    - ☐ Very satisfied
    - ☐ Satisfied
    - ☐ Not satisfied

……with my experience of learning about the research study and signing the consent form.

Anything else you would like to say (optional)?

Thank you for filling out this survey.
