# Supplementary material for: An evaluation of the process of informed consent: views from research participants and staff
Source: Trials. 2021 Aug 18;22:544. doi: 10.1186/s13063-021-05493-1 (PMC8371296; doi:10.1186/s13063-021-05493-1)
Supplement: Supplementary file 2 — Additional file 2. Research Staff Survey. [file 13063_2021_5493_MOESM2_ESM.pdf]

Research Staff Questionnaire

Have you facilitated an informed consent discussion for any kind of a research study, with a layperson, in Ireland or the United Kingdom?

- ☐ Yes
- ☐ No

If yes:

We are conducting a research study about how informed consent for research studies takes place. We would greatly value your input. The survey consists of 16 multiple choice questions, and will take about 10 minutes to complete.

1. Are you a (please select all that apply):
   - ☐ Principal investigator
   - ☐ Sub-investigator
   - ☐ Research nurse
   - ☐ Study Coordinator
   - ☐ Research Scientist
   - ☐ Research Allied Health Professional
   - ☐ Other – please specify:

2. Where do you facilitate informed consent discussions?
   - ☐ Primary Care / General Practice
   - ☐ Hospital
   - ☐ University
   - ☐ Other – please specify:

3. What kind of studies have you / do you work on (please select all that apply)?
   - ☐ Observational studies
   - ☐ Registry studies
   - ☐ Translational / Biomarker / Biobanking studies
   - ☐ Clinical Trials of Investigational Medicinal Products

- ☐ Non-Clinical Trial of Investigational Medicinal Product (surgery, radiation, psychology, physiotherapy intervention etc).
- ☐ Medical Device studies
- ☐ Other – please specify:

4. How much experience do you have facilitating informed consent discussions with clinical research / trial participants?
   - ☐ Less than 1 year
   - ☐ 1 - 2 years
   - ☐ 2 - 3 years
   - ☐ 3 - 5 years
   - ☐ 5+ years

5. Have you ever received training on how to take informed consent from a research participant?
   - ☐ Yes
   - ☐ No

If yes, was this (please tick all that apply):

- ☐ Formal, structured training (seminar, training day or session)
- ☐ Informal (verbal instruction or feedback while in clinic)
- ☐ Observation of a senior colleague
- ☐ Other – please specify:

6. How do you normally facilitate informed consent discussions (please tick all that apply)?
   - ☐ Explain study verbally to the patient
   - ☐ Give the patient an information leaflet and ask them to read it
   - ☐ Read the information leaflet to the patient
   - ☐ Show the patient a video or website about the study
   - ☐ Other – please specify:

7. How confident are you, generally speaking, that your patients have understood the trial / research study, after your explanation of the study and / or they have read the

information leaflet?

- ☐ Very confident
- ☐ Confident
- ☐ Somewhat Confident
- ☐ Not very Confident
- ☐ Not at all Confident

8. What do you find difficult about facilitating informed consent discussions (tick all that apply)?
   - ☐ Not enough time; time pressures in clinic
   - ☐ Difficult to explain complex information (disease related or study methodology related)
   - ☐ Patient is anxious or upset
   - ☐ Have dual role of health care professional and researcher
   - ☐ Information leaflet & consent form is too long and / or too complicated
   - ☐ Difficulty for patients to understand the information
   - ☐ Other – please specify:
   - ☐ Not applicable; no difficulties

9. Do you think the patient information leaflets and consent forms are in general:
   - ☐ Very easy for patients to understand
   - ☐ Easy for patients to understand
   - ☐ Fairly easy for patients to understand
   - ☐ Fairly hard for patients to understand
   - ☐ Very hard for patients to understand

10. What situations make you less likely to offer a patient the chance to participate in a study or trial (tick all that apply)?
    - ☐ You don’t have enough time in clinic
    - ☐ Patient is too anxious or upset
    - ☐ You don’t think patient will understand the study
    - ☐ You think the patient has already received too much information at this visit

☐ Patient doesn't have enough time

☐ Other – please specify:

11. What would help you improve the informed consent process for patients (please tick all that apply?).

    ☐ More time for you to spend with the patient

    ☐ A shorter and / or simpler Patient Information Leaflet & Consent Form

    ☐ Resources such as an App or video

    ☐ More time for another member of staff (research nurse, study coordinator, investigator) to spend with the patient

    ☐ A Patient Information Leaflet with simple diagrams or pictures

    ☐ Other – please specify:

    ☐ Not applicable; feel no improvements necessary

12. Do you use a structured approach to providing information to potential study / trial participants? E.g. use a checklist or follow the layout of the Patient Information Leaflet?

    ☐ Yes, all the time

    ☐ Yes, often

    ☐ Yes, Occasionally

    ☐ No, never

13. Once consent is obtained and the study commenced, do you continue to monitor consent received? i.e. do you approach participant(s) at different stages throughout the duration of the study to ensure continuous consent (i.e. that the participant is still happy to take part in the study)?

    ☐ Yes, all the time

    ☐ Yes, often

    ☐ Yes, Occasionally

    ☐ No, never

14. Overall, how confident do you feel in your ability to facilitate a good informed consent process?

- ☐ Very confident
- ☐ Confident
- ☐ Somewhat Confident
- ☐ Not very Confident
- ☐ Not at all Confident

15. Do you normally check a patient's understanding of a study / trial?

- ☐ Yes
- ☐ No

If yes, how (tick all that apply)?

- ☐ Ask patients if they have understood
- ☐ Ask patients to teach back or talk back the information
- ☐ Encourage patients to ask questions
- ☐ Other – please specify:

16. Approximately what was the duration of your **last** discussion of a study / trial with a patient?

-------- hours --------- minutes

Anything else you would like to say (optional)?

Thank you for completing this questionnaire.
