# Supplementary material for: An evaluation of the process of informed consent: views from research participants and staff
Source: Trials. 2021 Aug 18;22:544. doi: 10.1186/s13063-021-05493-1 (PMC8371296; doi:10.1186/s13063-021-05493-1)
Supplement: Supplementary file 3 — Additional file 3. Participant Information Leaflet (Research Participants). [file 13063_2021_5493_MOESM3_ESM.pdf]

Participant Information Leaflet

Study Title: A study of the process of informed consent from the perspectives of clinical research participants and clinical research staff.

Principal Investigator of study: Prof Peter Doran

Principal Investigator for SVUH: Dr Rachel Crowley

We invite you to fill out a short survey for a research study. Thank you for reading this leaflet.

What is the aim of this study?

The aim of the study is to find out the views of people who have taken part in research studies in Ireland or the United Kingdom (UK) in the past. Research staff do their best to explain a research study to participants, but studies can be difficult to understand. We want to know how satisfied you were with the information and time you were given before you agreed to take part in your previous research study. We also want to know how comfortable you were asking the research staff questions. We hope that your views will help researchers to explain research studies better to people in the future.

Why have I been asked to take part?

We are asking you to fill out this short survey because you agreed to take part in a research study in the past in Ireland or the UK.

What do I do if I want to take part?

If you want to take part, please fill out the short survey attached to this information leaflet. The survey has 14 'tick-box' type (multiple-choice) questions. It will take 5 to 10 minutes to fill out. If any of the questions make you uncomfortable, you don't have to answer them. However, we would be grateful if you could fill in as many questions as you can so that we get as much information as possible for the study.

Where do I return the survey to? [Paper-based survey only]

Once you have filled out the survey, please put it into the stamped address envelope we have given you and post it. By returning the survey by post, you are agreeing to take part in this survey study.

Will my taking part in this study be kept private?

Yes. The survey will not ask for any of your personal information (such as your name, address, date of birth, hospital number).

[Paper-based survey only] Please do **not** write any of your personal information on your survey.

[Online survey only] By filling out this survey online, you are agreeing to take part in this survey study.

Who should I contact for further information about this study?

You can contact: Lydia O'Sullivan, study coordinator

Phone: 086 176 0116 Email: lydia.osullivan@ucd.ie
