# Supplementary material for: An evaluation of the process of informed consent: views from research participants and staff
Source: Trials. 2021 Aug 18;22:544. doi: 10.1186/s13063-021-05493-1 (PMC8371296; doi:10.1186/s13063-021-05493-1)
Supplement: Supplementary file 4 — Additional file 4. Participant Information Leaflet (Research Staff). [file 13063_2021_5493_MOESM4_ESM.pdf]

Participant Information Leaflet (Research Staff Members)

Study Title: A study of the process of informed consent from the perspectives of clinical research participants and clinical research staff.

Principal Investigator of study: Prof Peter Doran

Principal Investigator for SVUH: Dr Rachel Crowley

We invite you to take part in a short, anonymous survey for a research study. Thank you for reading this leaflet.

What is the aim of this study?

The aim of the study is to find out the views of research staff who have facilitated informed consent discussions with research participants in Ireland or the United Kingdom (UK). Studies have indicated that facilitating informed consent discussions with research participants can be challenging. We want to find out the perceptions of research staff.

Why have I been asked to take part?

We are asking you to fill out this short survey if you have facilitated informed consent discussions with research participants in Ireland or the UK. This includes any kind of research.

Do I have to take part?

No. You do not have to fill out this survey.

What do I do I want to take part?

If you want to take part, please fill out the short survey attached to this information leaflet. The survey has 16 multiple-choice questions. It will take 5 to 10 minutes to fill out. If any of the questions make you uncomfortable, you don't have to answer them. However, we would be grateful if you could fill in as many questions as you can so that we get as much information as possible for the study.

Will my taking part in this study be kept private?

Yes. The survey will not ask for any of your personal information (such as your name, address, date of birth, where you work etc). Please do **not** write any of your personal information on your survey. By filling out this survey online, you are giving your consent to take part in the study.

Who should I contact for further information about this study?

You can contact: Lydia O'Sullivan, study coordinator

Phone: 086 176 0116   Email: lydia.osullivan@ucd.ie
