# Supplementary material for: An evaluation of the process of informed consent: views from research participants and staff
Source: Trials. 2021 Aug 18;22:544. doi: 10.1186/s13063-021-05493-1 (PMC8371296; doi:10.1186/s13063-021-05493-1)
Supplement: Supplementary file 6 — Additional file 6. Complete list of responses from research participants to open-ended question [file 13063_2021_5493_MOESM6_ESM.pdf]

**Additional File 6 – Complete list of responses from research participants to open-ended question**

Please note that quotations are verbatim except where it was necessary to redact some words to ensure participant confidentiality.

1. I sought out studies. I wish as a patient I didn't have to seek. I share as much as I can and others are astounded x,y and z are out there. There are not enough databases or work being done on databases in Ireland. Every person with Arthritis should be in one. There will never be cures if Researchers don't have detailed info. via databases. Please please please lobby the government for funding. Best of luck with your research but unless you get all public and private consultants to work with you and patients and patients as equals and have databases you are peeing into the wind.

2. I also work in research so was very keen to participate in study, which meant the research didn't have to spend long explaining the study too me but they whole way throughout they gave me opportunity to ask questions and think about participation if i need to.

3. I had about three discussions last one face to face. All were spaced by a few weeks.snd so my confidence, increased familiarity and ultimate agreement with [Name of Principal Investigator] was comfortable. Communication is paramount.

4. There should be more staff available to help weight loss and not just be dropped by the research clinic when you don't reach weight loss target. The system fails you. It's very disheartening to people.

5. Very clear and concise - all stages explained.

6. It was an interesting experience.

7. See below. I have included my work email below, should you have any further Qs if I can be of any further assistance (not cost!). As a qualified lawyer completing this survey, I fully understand how difficult it is to ? 1) information overload; and 2) ensuring you have produced sufficient information so as to ensure that any consent provided was fully informed consent. Suggestion ? would be to have 2 ? distinct and separate stages: 1) provision of consent form, ? of study etc & an explanation, Q session between patient & doctor / research nurse / nurse at a general level. 2) a specific 2nd visit / appointment would then take place, whereby the patient could discuss the content of the consent form, and ask any further questions following after own research and or reflections / consideration of study.

8. Taking part in the research trial allows me to access a much needed drug that was not available in Ireland at the time.

9. They emailed the document to me before went for study visit. This was really helpful to consider info in my own time in my own surroundings. This meant time could be

spent asking for clarification on areas of concern during the meeting without feeling rushed in any way.

10. Everything seemed rushed. The leaflet which I understood due to education but it wasn't clear or simple.

11. I found the information leaflet very long and it was not obvious how certain parts would apply to me.

12. Friendly staff I trust are the most important factor for me.

13. Your question up is parsed in such a way as to imply a person might only have participated in one research study.

14. As a health care worker I was inspired to take part in any "non invasive" clinical trials and would encourage anyone to give it consideration.  Consent is key....this is the part participants must understand.

15. All studies that I have taken part in have been very well explained and all questions covered professionally.

16. Probably no harm to get the information in advance of researcher chatting to you.

17. Need more time with the headset and would like to have an appointment with hypnotherapist so that I can stop forcefully eat.

18. No, very happy with information given and explained.

19. Thank you for the opportunities you've given me to take part in these trials.

20. I'm a research staff. ?bias? - Understanding - Time needed to consent. Overall - very good experience.

21. Very happy with my research nurse.

22. The research nurse was very friendly, patient and professional. She paused regularly while reading and explaining the information and gave me plenty of time to ask questions. I am taking part in the drug trial and the research nurse has continued to listen and respond to queries. I am very pleased with every aspect.

23. I am extremely happy with all the staff I have come into contact throughout the study.

24. [Name of Nurse], [Name of Hospital] is excellent, very helpful and so pleasant to deal with. She explains everything clearly and makes sure all your questions are answered. I know she's always on the end of the phone if needed, which gives me great peace of mind. 10/10

25. I feel lucky to be taking part in the trial. Thank you all.

26. The research staff were very encouraging and open. I felt very involved in process.

27. It's all down to confidence. [Name of doctor] showed he was confident that this trial was the optimum choice for me. His nursing staff made it personal, that they cared and were interested in me, and were easily contactable. They were very familiar with the trial and supported me so well. I just had to keep the body in good order to receive/accept the treatment.

28. The staff were great.

29. The staff are lovely and always have time for me and explain anything that I don't understand. I always feel very comfortable.

30. I found the staff to be kind, efficient and explained everything to me as I had my procedure done.

31. I would like to say the research staff really made me feel at ease and were very helpful.

32. Would like to know how initial results of trial going.

33. Found the research staff very nice to work with.

34. Studies should use Consentic to do the consent. Much better than using research staff to explain the research, and able to be emailed which is important in covid19

35. Never got the results of the 10 year study.

36. It was for the Common Cold Research.

37. I developed Addison's desease from trial drug. No back up when I had to discinue trial due to side effects of trial drug.

38. I am open to research as I feel I could make a difference.

39. I asked for a copy of the final research results as I was interested in the results of the study (getting a copy was an option on the consent form I think), but I never received it, maybe it took longer than expected.

40. I took part in a study for non invasive prenatal test. I found it really good. I was willing to give as much access as necessary to the company because of the quality of information that was given. I let them have access to my placenta and to my child heel prick test because I knew the benefits for future generations.

41. Would prefer to have known before i visited hospital that i was going to be asked to participate.

42. I was asked a mental arithmetic question which I was never good at I was actually very good at written maths. I felt a bit inadequate on account of not being good at the mental arithmetic.

43. It was pre and post major surgery and there was blood tests and biopsies from removed body parts from surgery. Post surgery blood tests.

44. It's a wonderful thing to be able to help by taking part in these trials.

45. Very happy to take part in any trials.

46. I have taken part in a few research studies and was delighted to have played a part in research.

47. Staff asked for my umbilical cord for research after an extremely traumatic birth while I was 22 and a single mother. I though everybody did that. That was 32 years ago.

48. Because of the subject matter and where I was asked about the study, I didn't have enough time to answer all the questions.

49. The nurse who did my study was so professional but also made me feel comfortable and made me at ease so I didnt fear asking a question. She made sure I understood exactly what the study was about in language that I understood perfectly.

50. I was never contracted about it after.

51. I would have liked some feed back from the researchers.

52. Did 2 studies, answers may not be exactly appropriate for each trial.

53. My experience was that the researchers were kind friendly and professional.

54. Research doctor was very nice and professional. Got valuable information on my health.

55. I still am being observed about my trial.

56. I was focussed on my output and outcome more than the study itself.

57. This study was many years ago (1980's) and I was recruited alongside students on my course to be control subjects. We weren't really told what the researcher was

exploring. But it only involved running on a treadmill and cycling whilst all wired up, so we didn't mind. Retrospectively we certainly should have been better informed.

58. I had a good experience had a brain scan as well. Everyone very helpful. Had to go to Dublin from Galway and got train paid along with food.

59. I was on a trial concerning ovarian cancer. I started in [date]. I was followed up until [date]. The cancer recurred in [date] and I began chemotherapy again in [date]. I've asked about the trial and been told I'm no longer on it. I have had no contact from the trial team to officially tell me this or explain why. The trial was [Name of trial].

60. The information I had to provide was extensive and needed more time to answer questions.

61. Very pleasant research staff.

62. It was research into Alzheimer's in Trinity College Dublin over 10 years ago. I volunteered.

63. Was not giving any feed back in the research i had taken part in.

64. I was not asked to sign a consent form.

65. Did not sign a consent form just asked to give a blood sample as they were doing research on the type of cancer I have.

66. It was over 20 years ago.

67. Secrecy was paramount- drug in development.

68. The research study was introduced during a hospital/clinic appointment. I think that a prior notification that this would happen would have been useful. Normally at a hospital appointment, I would already have questions to ask and information to clarify. So the additional information about research can be difficult to process on day. Prior notification would allow the patient time to mentally repair, and on a practical note allow them to allocate extra time for hospital visit.

69. Doing the trial was worth the effort.

70. Due to covid pandemic and pre assessment group that would normally have take place before my type of surgery was cancelled. So I felt I hadn't opportunity to have all information I needed. Pre pandemic patients would have normally met as group and opportunity to ask q about procedures discussed.
