# Supplementary material for: An evaluation of the process of informed consent: views from research participants and staff
Source: Trials. 2021 Aug 18;22:544. doi: 10.1186/s13063-021-05493-1 (PMC8371296; doi:10.1186/s13063-021-05493-1)
Supplement: Supplementary file 7 — Additional file 7. Complete list of responses from research staff to open-ended question [file 13063_2021_5493_MOESM7_ESM.pdf]

Additional File 7 – Complete list of responses from research staff to open-ended question

Please note that quotations are verbatim except where it was necessary to redact some words to ensure participant confidentiality.

1. Research nurses generally do every consent except for Clinical Trials , for academic intervention and non intervention consent - the same principals apply and for CT , nurses generally spend a lot of time explaining the PIL after it has been signed by doctors - unless an allocated time and space are available but busy clinics are not ideal.

2. While a short video is an excellent idea for some patients, I feel the length would be an issue and some patients are less likely to focus on an animation/video compared to a conversational style explanation of the study where they can interrupt and ask questions at any time. Volume of the video would also have to be adjusted to make sure hard-of-hearing patients can hear but at the same time, patients in the cubicle next to them do not hear as they might get concerned. I think it's important to make sure the patient goes home with a copy of the participant information sheet and informed consent document as questions may not arise during the taking of consent process itself.

3. As a researcher, it feels like the definition of informed consent is constantly changing, the bar is always going up. This is of course, a good thing. But the consequence is the need to ongoing dialogue. This requires significant resources that the system is not currently providing. So, at the moment, the net results of increased standards in consent is less research/smaller study sizes + destruction of datasets = less robust results.

4. The informed consent process isn't a once off meeting . Generally for complex studies one will meet with patient a couple of times/ take and receive calls re study from patient and relatives and also meet with close relative.

5. Info regarding GDPR in the Rep of Ireland is much too lengthy and repetitive and needs to be massively reduced. Often the length of an info sheet is enough to put a patient off. We need greater facilitation of remote consent (telephone etc.) especially with lack of visiting due to COVID.

6. The discussion/explanation 5-8 mins. One must get related to the person first before launching in to the research so getting related, explaining the study could be up to 15 mins. A complete consent process is not just the discussion alone so the entire consent process could take longer as it includes time for the patient to read the PIL, address questions, take written consent, photocopy the PIL and consent, write up

the patients chart. this takes time. Also having photocopy facilities to make a copy for the patients - sometimes these resources are not easy to access or not available.

7. PILs much too complex particularly with data protection which patients find cumbersome and excessive.

8. Informed consent can be conducted very differently depending on the person that delivers it. I think language that is used is very important. I also think that patients should have a basic understanding of the standard of care treatment *properly* before being approached and discussed about a clinical trial as often you are introducing another variable that can cause confusion. I also find that patients often look for statistics or probable outcomes/previous studies/reasoning etc and research staff should have more formalised training in the trial and consent process. Research staff are often struggle for dedicated space to conduct informed consent and this can add unnecessary stress and burden to the process. Clinical trials personnel should have dedicated areas for completing this important process with appropriate resources and time availability.

9. I welcome the review of PIL and consent forms so that they are written in plain English, without jargon or complex information and diagrams or pictures would be excellent.... they are generally not user friendly from a patient perspective, just a legal way to impart necessary information.

10. Time is always an issue and the complexity of studies now can turn patients off. that is extra appointments for questionnaires and translational samples bloods that are required.

11. It would be a great jump to see research staff be able to perform the informed consent process themselves independently.

12. Resources badly needed. Dedicated trial clinics. Protected time.

13. Key educational role for those recruiting and taking consent; some cultural influence.

14. Discussing consent in a busy clinical environment is very difficult. I usually discuss the study and ask the patient to read the PIL and I will get back to them (2 weeks) for further discussion at which point I will focus on their further understanding of consent.

15. would always emphasis that participation is voluntary and if they are unhappy at any time with participating that they may withdraw. Would give them the subject information leaflet to take away and arrange to contact them regarding the study when they have had adequate time to read and discuss study with members of family.

16. Use of patient teaching evaluation tool at the end of consenting.

17. I was recruiting older patients with complex multimorbidity to a cluster RCT. Participating GP practices identified and started the consent process. The intervention was a GP provided (their usual GP) medication review and so any risk to the patient from participating was very low. Patients who wanted to discuss further were directed towards me (study manager- GP and PHD student). However a conversation with their own GP was preferred. The PIL and consent forms followed a format that was required by the ethics committee. It was overly complicated in my opinion. 1 in 4 Irish adults have impaired literacy skills, and I imagine would be much higher in my cohort. I think the amount of paperwork was off putting for patients and a deterrent to them in participating. Some GPs identified this and said to me that their patients would be embarrassed about this. Many patients ticked the wrong boxes on a consent form that was too long and complicated- when I telephoned to clarify if they indeed wanted to consent- they reported being confused by the form. I think this is a major issue for pragmatic real world trials that want to include older, frailer, multimorbid patients.

18. Info leaflets are getting more complicated with GDPR/data protection information. It is almost impossible to make it shorter without risking rejection by ethics committee.

19. Ethics boards often require information sheets and consent forms to be in a particular template. It can be really hard to design a patient friendly information leaflet within these constraints.

20. The amount of info required on the PIS by ethics committees is excessive and intimidating and hampers research recruitment and participation....and ultimately out ability to know how best to care for patients.

21. Length of time for a consent discussion will depend on the complecity of the study and the risk assocaited with participation. 5 minutes is sufficient for taking consent as part of an interview only based study, but would not be sufficient for a CTIMP.

22. Is it feasible and practical for the participant? Distance from where trial is happening, commitments at home, ability to drive or travel, personal circumstances all need o be taken into account.

23. Reconsent due to continually updates to PIL even for patients who have moved to another treatment is a prolonged and poor process as pateints end up resigned original templates with additions although they are no longer on active tx., very patient unfriendly.

24. Informed consent Part 1 explaining study may be complex but generally easy to explain and answer questions. Part 2 of consent outlining GDPR, Storage of Data, inside and outside of Europe, storage and destruction of specimens, tends to be very long and boring for patients. Having 3+ signing sections and loads of boxes to initial as well as providing signatures, is repetitious.

25. I feel the studies I have done are low risk - the main risk concerns patient data retention.

26. Often, patients want to defer to doctor rather than really listening "Sure, if you think I should be in the study, I will."   " if you'd go in the study, if you were in my position, I will".

27. Study will hopefully lead to important outcomes to help patients understand the key messages. The "legalize" should completely removed.

28. The issue of informed consent, and electronic consent, is increasingly relevant with the Covid-19 pandemic. The issue of consent arises with the childhood flu vax, a novel vaccine in Ireland. BEST WISHES WITH YOUR RESEARCH.

29. I think it is really challenging to walk patients/parents through the informed consent process if all stages/processes of the research are to be explained properly and being fully aware of trying not to coerce in any way. I commit myself to this but then there can be reduced rates of recruitment which I then have to justify. I have had many challenging discussions with collaborators around rates of recruitment which may be lower than others but at least I know I am running my studies with the highest ethical standards....it can be very hard though!

30. Thanks for trying to improve the consent form process. Consents are generally difficult for patients to understand and more time should be given for the patients to process the information. Patients do not realize the number of extra visits or blood samples to be taken until later on in the process.
